# Assessing Healthcare Equity in Orthopaedic Surgery: An Analysis of Over 24,000 Surgical Cases

**DOI:** 10.5435/JAAOSGlobal-D-24-00240

**Published:** 2024-09-16

**Authors:** Zachary R. Visco, Ezan Chaudry, John S. Hudson, Moe R. Lim

**Affiliations:** From the Department of Orthopaedic Surgery, University of North Carolina School of Medicine, Chapel Hill, NC.

## Abstract

**Introduction:**

Health disparities have been widely studied in the primary care and surgical settings. The purpose of this study was to examine surgical access disparities for orthopaedic surgical cases performed at a large academic health center by comparing the relationship between patient demographic factors and surgical wait time.

**Methods:**

A total of 24,778 orthopaedic surgical cases from 2018 to 2022 at a public, tertiary care, Level I trauma center were retrospectively analyzed to assess for surgical timing disparities based on patient-specific factors, including race, sex, language, and socioeconomic status.

**Results:**

Elective surgical cases were completed with an average surgical wait time of 28.11 ± 26.34 days. Urgent surgical cases were completed with an average surgical wait time of 1.23 ± 1.50 days. Patient race, sex, language, and socioeconomic status had no effect on surgical wait time for urgent case scheduling. Female patients had longer average wait times in elective cases, whereas race had a weak association with increased wait time. Two-factor interaction analysis showed no multifactorial effects of patient demographic factors on surgical wait time. Patient race and socioeconomic status were associated with increased distance from surgical sites, although increased distance did not correlate with increased surgical wait time.

**Conclusion:**

Patient demographic factors did not demonstrate clinically notable associations with surgical timing in this patient cohort, in contrast to previous studies demonstrating the effects of race and socioeconomic status on healthcare outcomes and access. Race and socioeconomic status did correlate with increased distance from surgical centers although distance from surgical sites did not correlate with surgical wait time. This contributes to previous literature on healthcare equity and indicates that surgical wait time may not contribute to the known healthcare inequalities seen in minority and marginalized patients.

Health disparities are widely acknowledged in the medical literature, particularly in the primary care setting.^1,2^ Numerous previous studies have identified key underlying patient factors, specifically race, sex, and socioeconomic status, which are associated with decreased health access.^2-4^ These disparities have also been linked to systemic factors including economic inequality and institutionalized racism.^5,6^ Together, these elements contribute to variable patient access to care, quality of care, and most importantly, patient outcomes.^2,3^ These primary care findings have been identified in the surgical literature as well, again related to race, sex, and socioeconomic status.^7^ This is of particular concern given that surgical care is a key component of a robust, comprehensive health system.^8,9^ It is therefore essential that surgical treatment be provided in an equitable manner and that patient access to care and quality of care is not limited by patient demographic or systemic factors.

Surgical care has undergone notable changes in the past few decades, with a transition to increased subspecialty care and minimally invasive surgeries.^10^ Surgical demand has continued to rise in combination with increased availability of surgical services. This has enabled patients to receive treatment for a wider variety of surgical conditions, often through less invasive procedures. Surgical health disparities manifest as decreased access to care, surgical delays, decreased quality of care, and increased risk of postoperative complications.^7,11^ Wong et al^12^ stratified state counties by healthcare rankings and utilization, which demonstrated increased rates of health complications in low-ranked counties compared with higher-ranked counties. Furthermore, the presence of healthcare disparities in the outpatient and preoperative phases of care may predispose patients to the increased likelihood of complications in the perioperative and postoperative periods.^7^ This leads to increased patient and health system costs, which is of particular concern given the rising cost of surgical spending nationally.^13^

Healthcare access disparities have been noted throughout both general surgery and the various surgical subspecialties, including the field of orthopaedic surgery.^10,14,15,16,17,18^ Given that these disparities are often based on patient demographic factors, clinicians and surgeons have a responsibility to identify strategies to create more equitable orthopaedic surgical care. It is also essential that healthcare organizations be critical of their internal structures because healthcare inequalities can often stem from system-wide inequities. Increased surgical wait time and limited surgical access have been associated with worse patient outcomes in both the general surgery and orthopaedic settings.^19^ It is therefore imperative that surgical access be provided equitably to ensure that patients have convenient access to safe, high-quality care, for both urgent and elective surgical procedures.

This project aims to examine disparities in orthopaedic surgical access at a large, tertiary care, academic health center by assessing the impact of patient demographic factors on surgical timing. This study seeks to understand whether patient-specific variables have an effect on a patient's ability to schedule surgery and the amount of time it takes for those patients to actually undergo surgery. It also informs whether there are systemic factors that limit surgical access to different groups of patients. While previous literature has provided targeted review of surgical procedures, this is one of very few studies to analyze system-wide surgical data.^12,19,20^ The key aim of this study was to identify health inequities in orthopaedic surgical care that can drive future quality improvement goals and serve as a model for other health centers.

## Methods

### Data Collection

A retrospective review of all orthopaedic surgical cases performed at four academic surgical centers from January 1, 2018, to June 30, 2022, was completed. The primary institution is located in a suburban area with multiple surrounding Level 1 trauma centers. Data were collected from the Carolina Data Warehouse for Health and Epic, resulting in an initial sample size of n = 24,778 cases. Cases were defined as procedures performed by orthopaedic surgeons at our institution. This study was approved as a retrospective review by the UNC Institutional Review Board, which waived the need to obtain informed consent. Patient demographic parameters were collected for each surgical case, including age, home zip code, sex, race, and primary language. Surgical case parameters were also collected for each case, including the date of the surgical case posting, the date of the surgery, and the relative urgency of the case posting. Urgent case classifications ranged from Class A (within 30 minutes) to Class E (within 48 hours). Elective case classifications ranged from Class F (within 1 week) to Class I (indefinite).

### Data Analysis

All data were analyzed using MATLAB Version 23b (MathWorks). Data were imported from a CSV file into MATLAB. Surgical cases were stratified into the respective orthopaedic service (eg, Orthopaedic Hand), if available, and were otherwise coded as “Orthopaedics”. Surgical cases were excluded from analysis if they had missing patient race, home zip code, sex, or language. Patient race was stratified into six groups: White, Black/African American, Asian, American Indian/Alaska Native, Native Hawaiian/Pacific Islander, or other. Patient primary language was stratified into three groups: English, Spanish, and other. Patients who chose not to list their preferred language or race were classified as other.

Patients were assigned an income quartile level based on the median income for their listed zip code using the 2020 Census data. Median household income by zip code was used as a proxy value for patient socioeconomic status.^1^ Patient's home zip code was also used to estimate patient's home distance from their respective surgery centers.^21^ A library of all US zip codes was downloaded from Simple Maps,^22^ which provides geographic information for each zip code as defined by the US Postal Service. Each zip code is represented by a single point, defined as the centroid or average geographic position of that region.^21,22^ The latitude and longitude of this point can then be calculated. The home zip code for each surgical patient was cross-referenced against this data set, and the estimated latitude and longitude for their home zip code were thus calculated. Three of the four surgical sites were within a half mile radius of each other and were treated as one location, given their close proximity. The geographic position (latitude and longitude) of this location and the fourth surgical site were identified. The distance between the patient's approximated home location and their respective surgical centers was then determined by calculating the spherical arc length between the geographic points and then calculating the linear distance between those points. This distance calculation assumed an average radius of the Earth of approximately 3,959 miles.

Surgical wait time was defined as the number of days between the date the surgical case was posted and the date the surgery was completed. The data were separated into elective and urgent cases. Urgent cases were defined as surgical cases that were posted with the intent of performing surgery within 48 hours. To control for variability in the data, an outlier analysis was performed on the surgical wait time. Both surgical wait time and patient distance from their surgical centers had notable skew, so outliers were defined as values more than five median absolute deviations away from the median. The surgical wait time outlier analysis was performed on the elective and urgent cases separately given the underlying assumption that the surgical wait time should be markedly greater in the elective group. Distance outlier analysis was performed using the urgent and elective cases in aggregate because the patient population was assumed to be the same between the two groups.

Data were then analyzed using an N-Way ANOVA to assess the difference in surgical wait time between the urgent and elective patient groups based on the above demographic factors. A two-factor N-Way analysis of variance (ANOVA) was used to assess the interaction between patient demographic variables (eg, between race and sex). Statistically significant findings were further evaluated using a multicomparison test with Bonferroni correction.

## Results

A total of 24,778 surgical cases were imported into MATLAB for analysis. Surgical cases with missing case information were removed, resulting in a net sample size of 20,654 surgical cases; 4,563 cases (22.09%) were classified as urgent (within 48 hours) and 16,091 cases (77.91%) were classified as elective. Outlier analysis was performed on the urgent and elective case surgical wait times separately, leaving 4,475 urgent cases and 15,425 elective cases for further analysis; 12,880 patients (64.7%) were White, 4,347 (21.8%) were Black or African American, 2,067 (10.4%) were classified as other, 290 (1.5%) were American Indian or Alaskan Native, 280 (1.4%) were Asian, and 36 (0.2%) were Native Hawaiian or Pacific Islander. 22.7% of patients were in the lowest-income quartile, 50.7% of patients were in the middle two quartiles, and 26.6% of patients were in the highest-income quartile. 50.9% of patients were female, and 49.1% of patients were male. The cases that were removed had similar demographic distribution (3.0% American Indian or Alaskan Native, 0.8% Asian, 18.9% Black or African American, and 58.5% White; 51% were male and 49% were female).

### Urgent Case Analysis

**Figure 1:**
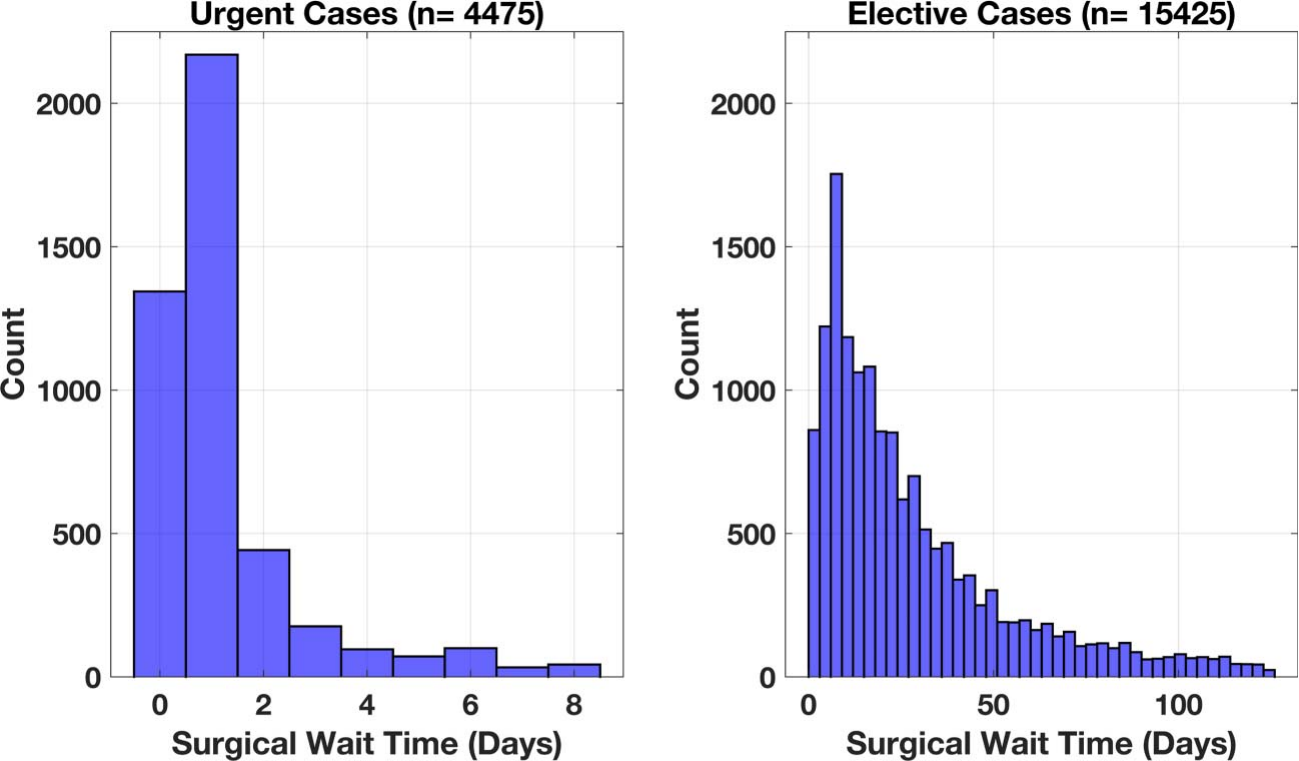
Graph demonstrating distribution of surgical wait times for urgent and elective orthopaedic surgical cases.

Urgent cases were analyzed with an N-Way ANOVA to assess the effect of race, language, income quartile, and sex on surgical wait times. Urgent cases had an average wait time of 1.23 ± 1.50 days (Figure 1). Language, race, sex, and income quartile did not demonstrate any notable relationships with surgical wait times. A two-factor interaction N-Way ANOVA was used to assess for interactions between the patient demographic factors, to determine their combined effect on surgical wait time. None of the interactions showed a notable relationship.

### Elective Case Analysis

Elective surgical cases were analyzed with an N-Way ANOVA in the same manner as urgent cases. Elective cases had an average wait time of 28.11 ± 26.34 days (*P* < 0.001; Figure 1). Race (*P* < 0.05) and sex (*P* < 1e-06) demonstrated a notable association with surgical wait times. Language and income by zip code did not demonstrate a notable association with surgical wait time. Subgroup analysis with Bonferroni correction was performed for each of the notable associations. Subgroup analysis based on race demonstrated a notable difference between Black/African American patients and Asian patients, with surgical wait times of 29.11 ± 26.03 and 23.95 ± 23.58 (*P* < 0.05). Female patients had average surgical wait times of 29.15 ± 26.58 days compared with male patients with wait times of 26.97 ± 26.03 days (p < 1e-06). A two-factor interaction N-Way ANOVA was used to assess for interactions between the patient demographic factors, to determine their combined effect on surgical wait time. None of the interactions showed a notable relationship.

### Zip Code Analysis

**Figure 2:**
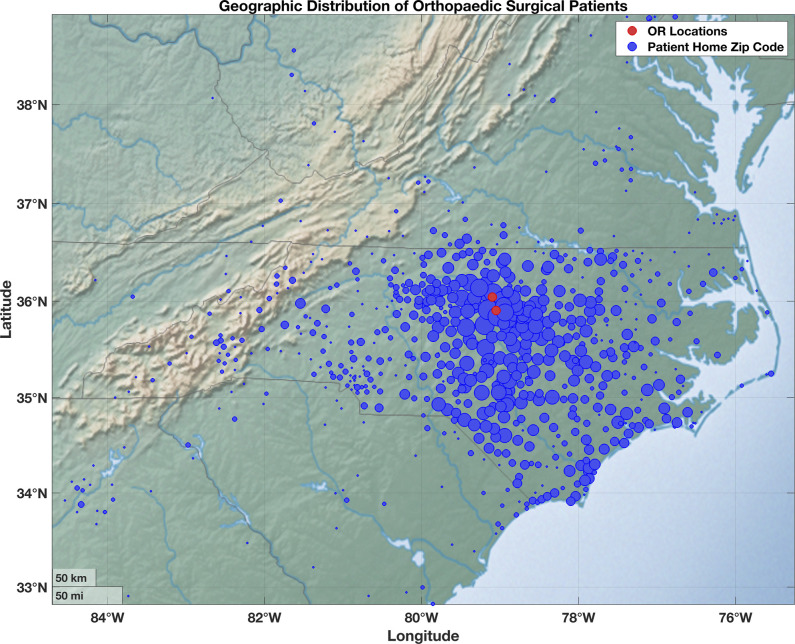
Geographic plot demonstrating the geographic distribution of patient home zip codes in the North Carolina region. Red dots indicate the locations of surgical sites used in the study. Each blue dot indicates patient home zip codes. The size of each blue dot indicates the number of patients who presented from each of those zip codes, with logarithmic scaling applied.

**Figure 3:**
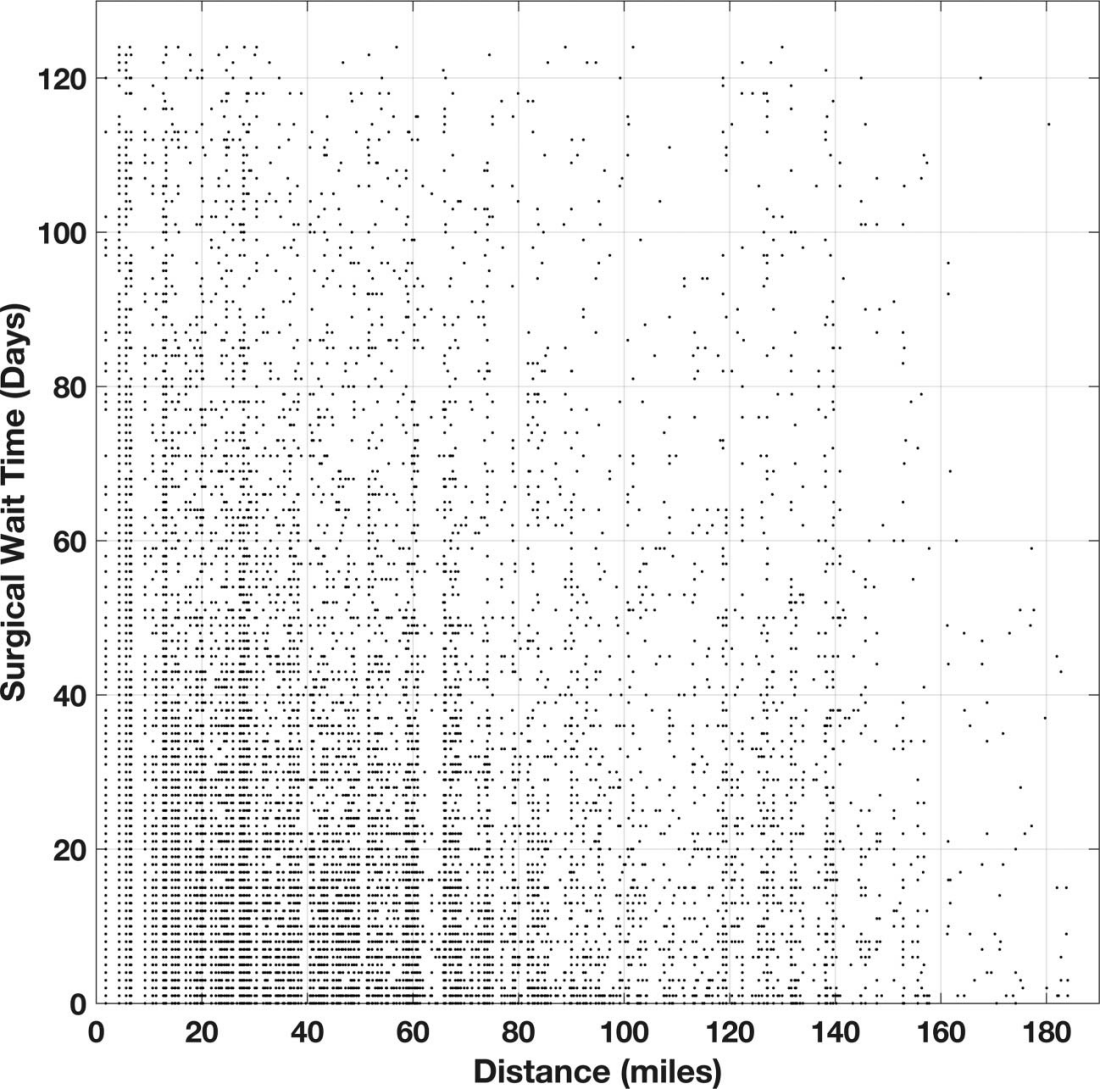
Scatter plot comparing patient distance from surgical centers with each patient's respective surgical wait time (R^2^ ≈ 0).

Patient home locations were approximated using their zip code, which was converted into latitude and longitude coordinates (Figure 2). An outlier analysis was performed on patient distance from their respective surgical sites, leaving 19,338 cases for further analysis. Patients lived an average of 39.56 miles ± 35.59 miles from their surgical centers. Patient location from their respective surgical centers showed no association with surgical wait time (Figure 3; R^2^ = 0.0012). Urgent surgical patients lived 40.70 ± 33.50 miles from their surgical sites while elective surgical patients lived 39.26 ± 36.16 miles from their surgical sites (*P* < 0.05). An N-Way ANOVA was then used to assess the association between distance and these patient demographic factors. Race (*P* < 0.001), sex (p < 1e-11), and income by zip code (p < 1e-05) demonstrated notable associations with distance from respective surgery centers. Language did not demonstrate any notable findings. Subgroup analysis with Bonferroni correction was performed for each of the notable associations. Subgroup analysis by race demonstrated a notable difference between White/Caucasian (36.71 ± 36.23 miles) and Black/African American patients (45.07 ± 33.45 miles; *P* < 0.01) and Black/African American and American Indian/Alaska Native (68.85 ± 28.32 miles; *P* < 0.05). Male patients lived an average of 42.72 ± 36.53 miles, and female patients lived an average of 36.57 ± 34.38 miles from their surgical site. Patients living in the highest-income quartile by zip code lived an average of 22.93 ± 28.30 miles from their surgical site while patients in the lowest quartile lived an average of 62.33 ± 31.49 miles away (*P* < 1e-06).

## Discussion

This study aimed to identify whether patient demographics had an impact on time to surgery for orthopaedic surgical cases at a large, academic, tertiary care medical center. It is widely accepted that healthcare access and quality are limited in certain patients. Independent patient demographic factors have been found to correlate with postoperative outcomes in a wide variety of surgical specialties, including orthopaedic surgery.^15,18,19,23,24^ Race and socioeconomic status are frequently identified as independent predictors of poor patient outcomes and increased complication rates.^17,20^ It is crucial that clinicians and surgeons understand their patient populations to understand which patients are at elevated risk of perioperative and postoperative complications. It is also necessary to understand which subgroups of patients have limited access to care, either because of independent factors or institutional barriers. This study analyzes data from a public, tertiary care, Level I trauma center with a diverse patient population to identify which of these factors contribute to orthopaedic surgical timing.

Most orthopaedic surgeries at this institution were performed as elective procedures. These were typically completed 1 to 4 weeks after the initial date of the surgical request. At this institution, case requests are placed by surgeons or residents and the cases enter a surgical depot. Surgical coordinators then place patients onto the operating room schedule based on available block time. Prior literature has demonstrated extensively that race and lower socioeconomic status are predictive of poor patient outcomes, as well as decreased surgical access.^12,20^ We initially hypothesized that minority patients, lower socioeconomic status patients, and female patients would have decreased access to care, which would manifest as longer surgical wait times. Race did demonstrate a weak association with surgical wait time, although subgroup analysis only identified a notable difference between Black/African American and Asian patients. Similarly, female patients had a longer wait time than male patients. These associations do support some previous literature, but the delay in surgical timing was quite small, and it is unlikely that this was a clinically notable difference for elective surgical procedures. Notably, there were no multifactor interactions when we considered the intersectional effects of patient demographics on surgical wait time. Previous studies have identified an increased risk of marginalization when patients are part of multiple marginalized groups, but this was not identified in our patient cohort.^25,26^ In addition, primary language and median household income by zip code showed no association with elective surgical timing in our patient cohort. This study was not designed to assess for postoperative outcomes and complications, but it does support previous work which identified increased surgical wait times in some marginalized patient populations.^20^

Urgent cases at our institution are typically completed as inpatient procedures after admission through the emergency department. This study did not identify any notable associations between patient demographic factors and surgical timing, which is in contrast to previous literature on the subject.^20^ This is encouraging given that it argues against systemic biases for scheduling and completing elective orthopaedic surgeries. However, this study did not assess the severity at the time of patient presentation for various surgical conditions. It is therefore possible that certain patient demographics had an increased rate of urgent surgical conditions, although this was not assessed as part of this study. Although urgent case timing was not affected by patient demographics, it is possible that patient demographics influenced the rates at which certain patients presented with urgent scheduling needs.

Socioeconomic status in the healthcare setting is a complex variable. It is related both to a patient's ability to access basic resources and to their ability to attain and maintain basic health needs.^3^ While this is multifactorial, it does stem in part from household income. Lower socioeconomic status and income have been consistently linked to higher morbidity and mortality, as well as decreased overall access to high-quality care.^3,27^ Furthermore, patients with higher socioeconomic status are more likely to adhere to postoperative protocols, which in turns leads to better postoperative outcomes.^27^ We therefore hypothesized that patients in higher-income zip codes would be associated with lower surgical wait times. Somewhat surprisingly, this study found that surgical wait times were independent of socioeconomic status. While this is a positive finding, it does suggest that other factors are contributing to poor patient outcomes in lower socioeconomic populations. This study did not consider the time it took for patients to schedule initial clinical appointments for elective case scheduling, so it is still possible that lower-income patients may have had difficulty establishing care. Future studies can assess the time necessary to establish care and the number of clinic visits necessary before scheduling surgery and then correlate this with surgical timing.

Patient access to health care has multiple components, including distance from clinical sites and access to reliable transportation. This influences a patient's ability to attend preoperative clinic appointments, to arrive on time for elective surgical scheduling, and to participate in postoperative therapy. This data set demonstrated that the patient catchment area for our healthcare institution is broad, but the four surgical sites considered in this study were both located in the central region of the state. We were concerned that patient distance from the surgical sites would have a notable correlation with surgical timing. However, this data set demonstrated that patient distance from their respective surgical sites had no impact on the time between surgical scheduling and surgical completion. As with socioeconomic status, this study did not consider whether patient distance correlated with the time necessary to establish initial clinical appointments. It is possible, and even likely, that patients living further from surgical sites were also living further from their clinic and therapy sites. Again, this is an area of future research to better understand how distance affects a patient's ability to access care and therapy.

Distance from surgical sites did not correlate with surgical timing in this patient cohort. There was a weakly notable difference in urgent versus elective surgical patients, but this was not practically notable since the difference was less than 1.5 miles. White patients lived almost 10 miles closer to their surgical centers compared with Black/African American patients on average and more than 30 miles closer to their surgical centers compared with American Indian/Alaska Native patients on average. Patients living in zip codes with the highest incomes were almost 40 miles closer to their respective surgical centers than patients living in the lowest-income zip codes, on average. Although this did not have any effect on orthopaedic surgical scheduling in this patient cohort, it does suggest that minority patient populations, and patients with lower income face an increased burden to access care. These patients have to travel further for their clinic appointments, emergency department visits, surgeries, and postoperative therapy appointments. This may not have an effect on the time needed to schedule surgery, but it does indicate that these patients spend more time traveling for appointments, more time out of work to access therapy, and may have increased difficulty reaching clinics or emergency departments when problems arise.

## Limitations

This study does have some limitations that warrant further discussion. This study was retrospective and therefore is unable to identify causal relationships between variables. There was a data attrition rate of approximately 20% because missing data, which may lead to selection bias due to incomplete patient sampling. In addition, this data set only includes 5 years of patient data and is a single institution data set. Although the sample size is large and the patient population is diverse in terms of race, sex, and socioeconomic status, this may limit the ability to generalize the information to other healthcare institutions because this is a single-institution study. This is of particular concern in smaller community hospitals with more homogeneous patient populations. In addition, the study design did not include information regarding time to schedule initial clinic appointments for electively scheduled procedures, which may have contributed to delays in surgical care. Finally, patient home location and socioeconomic status were approximated based on home zip code, rather than using exact addresses and income metrics.

## Conclusion

This study stems from previous work assessing healthcare equity in surgical fields, with a focus on orthopaedic surgery. Urgent orthopaedic surgical scheduling did not have any association with patient demographic factors in this cohort. However, we did identify some associations between race and sex with regard to elective orthopaedic scheduling, but this was not clinically significant. This study also demonstrated that distance from surgical sites was not related to surgical wait times in this patient cohort. Future studies are needed to further understand which patient demographics are most at-risk for surgical delays and complications, to build a more equitable healthcare system.
